# Design and Implementation of Cloud-Centric Configuration Repository for DIY IoT Applications

**DOI:** 10.3390/s18020474

**Published:** 2018-02-06

**Authors:** Shabir Ahmad, Lei Hang, Do Hyeun Kim

**Affiliations:** Department of Computer Engineering, Jeju National University, Jeju 63243, Korea; shabir@jejunu.ac.kr (S.A.); hanglei112233@hotmail.com (L.H.)

**Keywords:** Internet of Things, wireless sensor networks, smart space, cloud computing, configuration management

## Abstract

The Do-It-Yourself (DIY) vision for the design of a smart and customizable IoT application demands the involvement of the general public in its development process. The general public lacks the technical knowledge for programming state-of-the-art prototyping and development kits. The latest IoT kits, for example, Raspberry Pi, are revolutionizing the DIY paradigm for IoT, and more than ever, a DIY intuitive programming interface is required to enable the masses to interact with and customize the behavior of remote IoT devices on the Internet. However, in most cases, these DIY toolkits store the resultant configuration data in local storage and, thus, cannot be accessed remotely. This paper presents the novel implementation of such a system, which not only enables the general public to customize the behavior of remote IoT devices through a visual interface, but also makes the configuration available everywhere and anytime by leveraging the power of cloud-based platforms. The interface enables the visualization of the resources exposed by remote embedded resources in the form of graphical virtual objects (VOs). These VOs are used to create the service design through simple operations like drag-and-drop and the setting of properties. The configuration created as a result is maintained as an XML document, which is ingested by the cloud platform, thus making it available to be used anywhere. We use the HTTP approach for the communication between the cloud and IoT toolbox and the cloud and real devices, but for communication between the toolbox and actual resources, CoAP is used. Finally, a smart home case study has been implemented and presented in order to assess the effectiveness of the proposed work.

## 1. Introduction

Humans by nature have a strong tendency to learn things by doing and trying to do things by one’s self [1]. There are various drivers that push humans towards the Do-It-Yourself (DIY) approach. The major drivers for the DIY paradigm are creativity, simplification, extension, economic reasons and the need to control things [2]. Apart from these drivers, the recent advancements and innovations in DIY electronics are providing an opportunity for the masses to demonstrate their creativity. Systems on Chip (SoC), electronics development platforms and kits in the form of Arduino and Raspberry Pi are a huge inspiration for DIY. The simplicity and ease of development on these platforms are attracting more and more people towards DIY and, hence, enabling the general masses to express their creativity and genius.

According to a report from the International Telecommunication Union (ITU) in 2005, the “Internet of Things (IoT) will connect objects from the world, both in a sensory and in an intelligent way” [3,4]. IoT as it was perceived until the recent past had not been adopted by the masses [5], while the connections and devices in the IoT have grown in numbers since its advent [6,7], and with the realization of the Industrial Internet of Things (IIoT) and 5G, the number is expected to increase exponentially [8]. This means that end-users’ involvement in the IoT creation process is a crucial factor for its successful adaptation. According to the recently updated Gartner Hype Cycle [9], IoT is of great importance and presents the idea that IoT has come out of its imaginative and fictive stage [10] and now is considered as the real deal [11,12]. However, the end-users should be a part of the creation process while having the power to discover things [6,13], control it and effectively use the applications for smart environments [14]. The same idea is also presented by Xiao et al. [15].

In addition to the DIY driver, motivations and the need for massive end-users’ involvement for the realization of IoT, the current makers revolution [16] is inculcating a new version of the DIY culture. Connecting things, people and ideas together is conceived as the term “making” [17]. The Internet is providing the bridge between the makers and the masses. Online communities of people not only share their ideas and creations, but also have a chance to communicate and help each other on a larger scale [18], thus eliminating the structural and technological obstacles in their way [19]. DiY Smart Experience (DiYSe) [20] presents a DIY manifesto of 13 statements focused towards the developers who design and implement digital creation systems for end-users. The manifesto also highlights the relation of DIY IoT to the maker movement.

Inspired by the maker movement, many researchers perceive IoT as not just the implementation of some proprietary services for a specific goal, but in fact, as a vast ecosystem of billions of devices [21], which present themselves as atomic services on the Internet. These services must be available for the masses to utilize them to make their custom solutions. This concept is termed as the Do-It-Yourself (DIY) paradigm, and its main motive is to reduce the dependence of common people on proprietary products. In other words, DIY is the technique or method of creating, modifying or repairing something without the direct aid of experts or professionals. As IoT envisions a global ecosystem of connected devices providing services to people, the DIY paradigm of development has been suggested as the prime solution towards the global realization and implementation of IoT. In the DIY scenario, for instance, the same general population that utilizes the services provided by the IoT implementation will be able to develop the IoT application according to their own needs and requirements. It is, however, a concern that the general population lacks the necessary technical experience and specifically the programming skills to develop IoT-related applications. This is why alternative development strategies are being investigated to provide people with DIY type development environments through which anyone, regardless of their programming skill level, can develop applications based on their own requirements [22]. In a nutshell, the relationship between IoT and DIY or maker communities are best conceived of as “Embracing IoT instead of Circumventing it”, and this work is a step forward towards this vision.

Many researchers have put efforts in this regard and proposed various DIY-based architecture for easing up the development of IoT application and more specifically for enabling the general public to tinker with actual IoT resources to make a real IoT application; however, the DIY toolboxes in most cases use desktop technologies that would mean the configuration of the services composed would reside on the local system, and thus, there will be no way to access the configuration from any remote server, which in turn would mean that people shall not be able to control the devices outside their premises.

The contribution of this research is three-fold; firstly, we present a visual service designer with the drag-and-drop approach with the aim focusing on zero-programming customization of the functionality offered by remote and constrained IoT devices. The Constrained Application Protocol (CoAP) is among the most popular communication protocols for devices having limited resources. It is being standardized by the Internet Engineering Task Force (IETF) [23]. The proposed system utilizes CoAP resources as part of a CoAP server, which is implemented using the Raspberry PI board. Raspberry PI is the latest SD card size platform based on Linux distribution named Raspbian OS. The reason for selecting this platform is the growing acceptance of the platform for the development of IoT devices by the DIY community all over the world. These CoAP resources are shared by the CoAP server with the visual service designer via a CoAP proxy. The proxy is a Java implementation based on the Californium [24] framework, which facilitates the discovery of CoAP resources and provides libraries for the generation of CoAP commands. The designer represents the resources as virtual objects. The user uses the graphical representations of the virtual objects to design a service flow. The service design is sent back to the proxy in the form of an XML document where it is parsed to extract the information regarding the customized behavior of the remote CoAP device. The proxy then generates CoAP commands using the Californium framework in order to operate the devices according to the new functionality defined.

We believe that it is not far in the future when such IoT devices will be commonly available through the Internet, and the non-programmer users will require such a DIY interface to customize the functionality of those devices. The focus of this work is not how to implement CoAP devices and program them as is the case for several implementations discussed in this section. The focus is rather to enable the general public to easily access remote devices and, based on the resources and functional capabilities exposed by these devices, provide an intuitive representation to the users in order to customize device’s operations according to the users’ needs. To our knowledge, the proposed DIY system is the first ever step towards the behavior customization of remote CoAP devices using a visual interface.

Secondly, in addition to the idea of the DIY toolbox, the paper also proposes a novel approach to store and sync the output configuration process on the cloud platform to make it available for use and possible customization. The cloud-based configuration server also has the ability to control the appliances and devices from outside of the local premises by just logging into server and triggering a few HTTP requests using the virtual objects interface.

Lastly, the work also proposes and enables the general public to add a generic entity called the smart space. The smart space can be perceived as any IoT world comprised of connected virtual resources aiming to do something in a more useful and automated way. The idea of this smart space is delineated by a smart home-based case study, which is implemented as an example scenario for the smart space, and numerous activities and snapshots of the case study are presented and discussed.

The rest of the paper is organized as follows: Section 2 covers major contributions in the related areas and provides an overview of similar research contributions. Section 3 outlines the conceptual architecture for the proposed work and briefly discusses the process to achieve the goal. Section 4 presents the detailed design of the system and provides the interaction model to describe the sequence of operations. Section 5 discusses the implementation environment and the technology stack used for the components used in the work. Section 6 covers the major steps of the execution results and provides snapshots of the proposed system. Section 7 gives an overview of the implementation of the smart home case study and discusses the ways to interact with the IoT server. Section 8 highlights the significance of the proposed work and compares it with previous works. Section 9, finally, concludes the paper and identifies future directions.

## 2. Related Work

The IoT vision is the realization of a worldwide network of connected and inter-operable smart devices that can provide services to people. Although the individual technologies in terms of communication and devices have improved tremendously, the implementation and realization of such a complex network are still in their nascent stage. The end goal is to have plug-n-play smart objects that can be deployed in any environment with an inter-operable interconnection backbone that allows them to blend with other smart objects around them. Standardization of frequency bands and protocols plays a pivotal role in accomplishing this goal. To provide a high-quality user experience, we need to find ways to overcome the hurdles, which include coping with the heterogeneity of the hardware and mass involvement of the general public in the IoT development and adaptation process. The first issue is being abstracted out with the help of a middleware-based solution and service-orientation. The second issue is of greater importance because in our view, the realization of a successful worldwide IoT implementation is not possible without the involvement of the masses in the development of IoT, and this leads to the intervention of visual programming-based techniques; DIY, in other words.

Recently, there have been several efforts focused towards mass DIY in the fields of electronic device design, creation and programming. Some efforts are based on hardware boards, which come with electronic block modules for the general public to combine these blocks in any way they like and express their creativity by creating new and smarter things. Raspberry Pi [25] and Arduino [26] boards are some of the first and most popular efforts in this regard. These boards provide their own programming environment, and the users must be able to know programming languages such as Python or Java to write code for these boards. Microsoft .NET Gadgeteer [27] is another open-source toolkit for creating customized electronic devices by combining smaller electronic blocks onto a main board. The main board of the .NET Gadgeteer system has an embedded processor and sockets to connect simple plug-and-play Gadgeteer modules, which includes display, camera, networking, storage and sensors. The toolkit is based on the .NET programming language. NET Gadgeteer is aimed at exciting students about learning programming, electronics and design using an object-oriented environment of development.

SAM [28] is another Kickstarter [29] project, which provides blocked electronics modules, which can be used by inventors, creators, designers, and so forth, without any distinction of being a beginner or an expert in the field. The main theme of the SAM project is to combine hardware, software and the Internet. All the block modules in the SAM kit are wireless, and the Python language is used to program the modules. Not only is the DIY approach limited to basic hardware kits that can be combined as the user likes, but a recent trend is to investigate generic electronics with a higher degree of customizability and to involve the general public in the design and creation of such products, as has been investigated by Mazzei et al. [30]. The same idea is backed by many other studies such as Feki et al. [31], where DIY has been considered among the future trends in the field of IoT development. The study by Scott and Chin [32] provides an instance of the application of the DIY approach to IoT development based on low-cost Systems on Chip (SoC) as suggested in the previous paragraphs. A more recent application of the DIY IoT approach to digital agriculture in the form of crop growth monitoring and irrigation decision support, and so forth, is presented by Jayaraman et al. [33].

The Internet is a key player in the implementation and adaptation of IoT. The World Wide Web (WWW) and web services have also been used to provide a medium for makers or creators to share their inventions and creation. This way, they may be able to aggregate and reuse creations of other people to make more useful and smarter things. Pachube [34] is a web-centric service to aggregate streams of data “feeds” to acquire and store information related to different types of sensing devices and the data they produce over time. It also provides the capabilities of processing, integration and data visualization in the form of a collection of applications and is based on the idea of “triggers”. A trigger can be defined as the arrival of data from a resource (hardware or software), and in response, it can be forwarded to a specific URL based on some rule/condition or processed in order to activate some other triggers. In Pachube, the feeds’ integrations or triggers created by one person can be shared for use by others, enabling rapid development and creativity.

A more recent development came in the form of IBM’s Node-RED [35] with a focus to reducing the coding efforts and lowering the technical bar for the developers. Node-RED users wire together graphical nodes taken from a panel in order to create flows and then deploy these flows to get the results. The nodes in Node-RED represent devices, software platforms and web services [35]. The approach used by Node-RED is a better solution for enabling mass involvement in the realization of IoT, especially from a maker’s or creator’s perspective. Although these efforts have simplified the design and creation of things for the end-users, still, there is some level of experience required on the part of the developer. One way or the other, the developers must know how to program in order to use these hardware platforms or web services.

Glue.things [36] is another recent project that implements the concepts of device integration and real-time communication using the recent technologies of web sockets, MQTT and CoAP. The protocols are utilized on real-time data stream networks to allow mashups of the data streams, add actions, etc. The final mashups are deployable in a distributed environment. The system especially focuses on the composition of data streams from web services and IoT devices with web interfaces. The main aim of Glue.things is to utilize web technologies for providing interoperability platform with REST APIs, JSON data models, web sockets, etc. The mashup interface is based on Node-RED. Another important aspect of the project is the utilization of well-established open source technologies.

Super Stream Collider (SSC) [37] is another platform that helps enable everyone, from novice IoT users to expert programmers, to develop IoT applications in the form of near-real-time data streams. The web-based interface for SSC enables anyone to create their own mashups by combining linked data sources and linked streams to create resources that can be used as applications for IoT scenarios. The system supports the drag-n-drop technique with a SPARQL/CQELS query language based editor. As the platform is intended for large data acquisitions through streams, it utilizes cloud infrastructure for fetching the data, processing and dissemination of data.

As part of the OpenIoT project, a visual development approach has been presented by Kefalakis et al. [38]. The visual development tools are intended to be used as an Integrated Development Environment (IDE) for the support of the IoT application development lifecycle. The tools presented are based on a semantic IoT architecture and claim to be a minimal programming environment for IoT application development. They use a node-based user interface theme to allow the user to model service graphs and then convert them into SPARQL queries.

The OPEL software platform [39] is designed considering the following requirements. First, the IoT platform should be programmable with an easy programming language. In particular, the programming language should have high productivity, portability and extendibility to enlarge the IoT ecosystem. A good candidate is the JavaScript (JS) language. Second, the platform should provide high-level APIs for easy application development. The APIs need to provide several functions, including sensor and device management, communication, etc. Third, the platform should support multiple IoT applications. User should be able to install multiple applications with different functions, which can be executed concurrently. Then, the user can exploit multiple services on a single device. Lastly, the software platform should enable the companion IoT device to communicate with its host device (e.g., Android smartphone) and to be controllable via its host device.

Other tools like ThingWorx [40], Particle.IO [41] and Dweet.IO [42], as outlined in [22], are also used extensively, but they have more of a Platform-As-A-Service (PAAS) nature and do not offer much flexibility to the general public. These tools, in addition to that, are not open source, and thus, the source code cannot be tweaked if any custom-made requirement needs to be incorporated.

All these technologies, to the best of our knowledge, either fully focus on the traditional desktop paradigm or the fully web-based paradigm. We believe that the strength of both paradigms can be integrated to design an IoT product that is closer to an ideal product.

## 3. Proposed DIY-Based IoT Cooperation Architecture and Composition System

**Figure 1:**
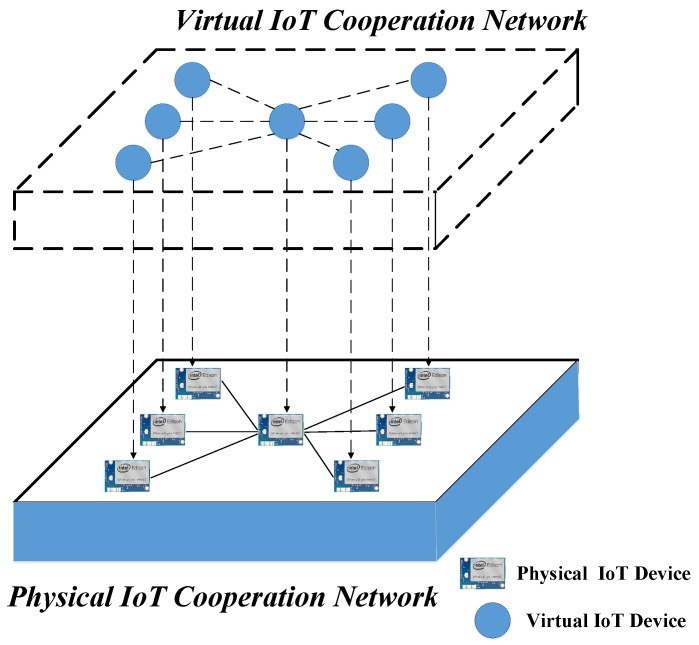
Mapping of cyber space to physical space.

The proposed IoT cooperation network as shown in Figure 1 mainly consists of the virtual and physical domain. The virtual IoT cooperation network provides a graphical interface for users to customize IoT applications in an intuitive way. The physical devices from the physical domain are represented as virtual objects that encapsulate the behaviors of the physical devices. These virtual objects are combined to generate service objects. Users utilize these service objects to customize the application process, which is further deployed to control the physical IoT network. The physical IoT cooperation network consists of various IoT sensors/actuators, and these devices receive and parse the deployment processing information from the virtual domain and then operate accordingly. For every device and composed service, an XML file is maintained, which stores the attributes and the URI of the server and the basic operation performed by the service. These XML files are stored on local storage initially as in other previous frameworks. In the next step, the XML data are ingested by the cloud-centric web platform and represented using web interfaces. The interfaces also provide better understanding of the virtual devices and the composed services. Moreover, the interface enables the user to control the devices associated with the platform, thus mitigating the need to go on premises in order to perform some action on the device.

**Figure 2:**
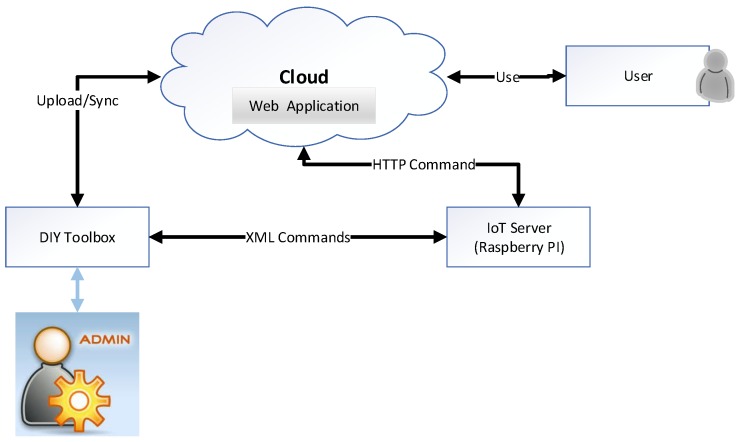
Conceptual architecture.

Figure 2 shows the conceptual architecture of our proposed work. The DIY toolbox shows the black box representation of the framework, while the IoT node is the actual physical things capable of communication. IoT things can be communicated offline with the IoT toolbox or can be controlled using cloud-based web interfaces if the client is not on the same system in which the toolbox is installed. The cloud ingests XML configuration data from the toolbox and represents them using the web interface for a better user experience for the client application. The cloud also has the ability to add some more configurations, and these configurations can be pushed to the IoT toolbox.

**Figure 3:**
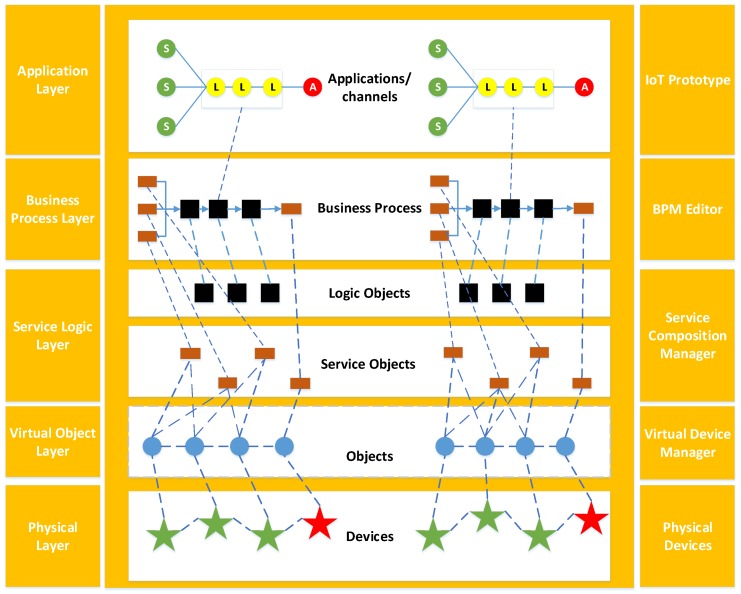
DIY toolbox layered architecture.

Figure 3 illustrates the white box representation of the DIY IoT toolbox conceptual view. The IoT toolbox is a layered architecture stacked together. Every layer has its own functionality and is decoupled from other layers, thus providing a modular approach.

The physical cooperation network layer consists of IoT sensors and actuators; for instance, the thermistor, which acts as a resister whose resistance varies significantly (more than in standard resistors) with temperature. This module’s output approaches 5 V as the temperature increases. As the temperature decreases, it approaches 0 V. Other examples could be humidity sensors and temperature sensors. Actuators can be fans, LEDs or any other physical things capable of performing some action. The virtual object layer represents the physical things in cyber space. The behavior of these objects is the action they perform, and the properties of these objects are the attributes associated with them. Virtual objects interconnect with each other on the service logic layer. This layer is responsible for providing an intuitive and easy to use visual environment where the VOs obtained from Virtual Object Manager (VOM) are rendered in graphical form. The users can drag-drop these graphical objects to compose various services. Two kinds of services can be composed. Firstly, is with the association of sensors and actuators only without the intervention of any logic object. Secondly is that logic objects like fuzzy control can be added between sensors and the actuator to develop more smart IoT applications. Once the services are composed, they can be connected using the Business Process Modeling (BPM) editor in the businesses process layer, and real-time application requirement are designed on this layer. Finally, the application layer specifies the communication protocol and interfaces used for the physical IoT prototype. This layer consists of three types of node: the sensor node, the proxy node and the actuator node. The sensor node is responsible for collecting environmental data from physical IoT sensors. The actuator node takes charge of operating the physical IoT actuators. The proxy node is in charge of supporting communication between sensor nodes and actuator nodes. Logic objects provide intelligent prediction and control services to operate the IoT actuators. The communication channel defines the specified communication protocol for data and command transmission.

**Figure 4:**
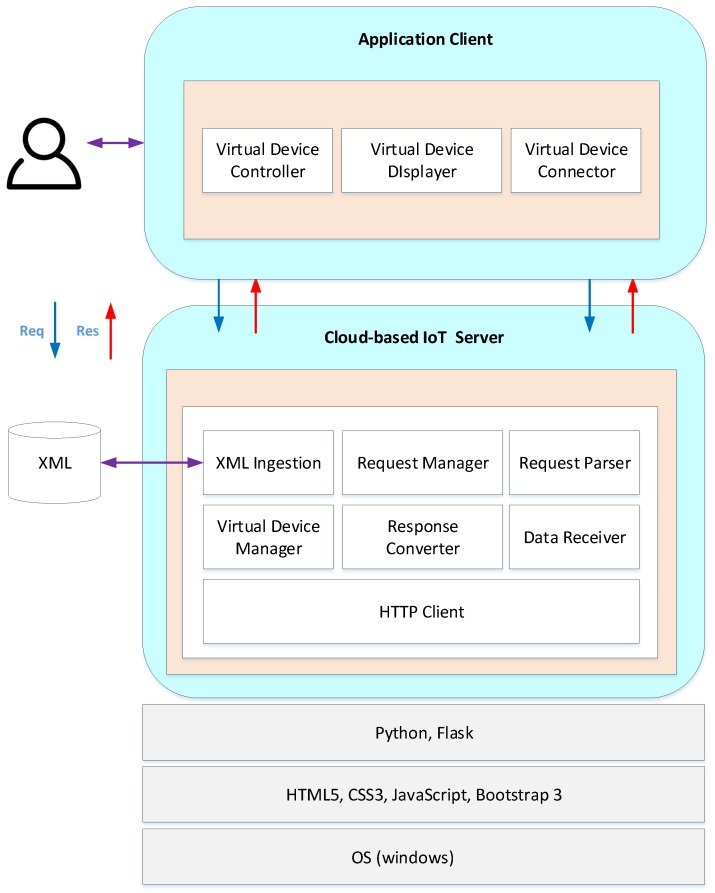
Cloud-centric application structure.

Figure 4 represents the block representation of the main components of the cloud-centric approach of storing the local XML configuration. The application is deployed on the Amazon Web Services (AWS) Compute Node which is also calledEC2 node using Python and Flask. We believe that using EC2 is a better approach because most of the application logic has been performed by the DIY desktop application, and the cloud only serves as a repository of XML files representing the already configured entity in a better UX interface. That being sad, using a full-fledged cloud platform like AWS IoT would be considered overkill. We used the Python Untangle library [43] for efficient parsing of XML data and converting them to respective objects that can be used by the Python-based web applications. The XML connector provides interfaces to connect the configuration data with the cloud-based web application, allowing CRUD procedures to run on the applications.

The virtual device manager provides functionality to manage the virtual devices. It is capable of creating the virtual devices, as well as connecting to the virtual devices. The virtual device manager provides a category that stores the virtual device information, and this category will be issued to the client whenever it receives the request from the app client. The request manager is responsible for accepting and processing the request from the application client. The request parser extracts the necessary parameters from the request and matches the related service to access the device. The HTTP client module is a separate communication module in a RESTful style, which is flexible and can be easily changed to adapt to different application protocols other than HTTP. The data receiver is in charge of receiving the response message from the IoT server. The response converter formats the response data, and after data formatting is completed, the processed data are visualized to the user on the web client. Moreover, for each virtual resource, there is an interface for controlling the state of the resource, and thus it could easily be controlled even if the user is not at his/her local premises.

## 4. Interaction Model

**Figure 5:**
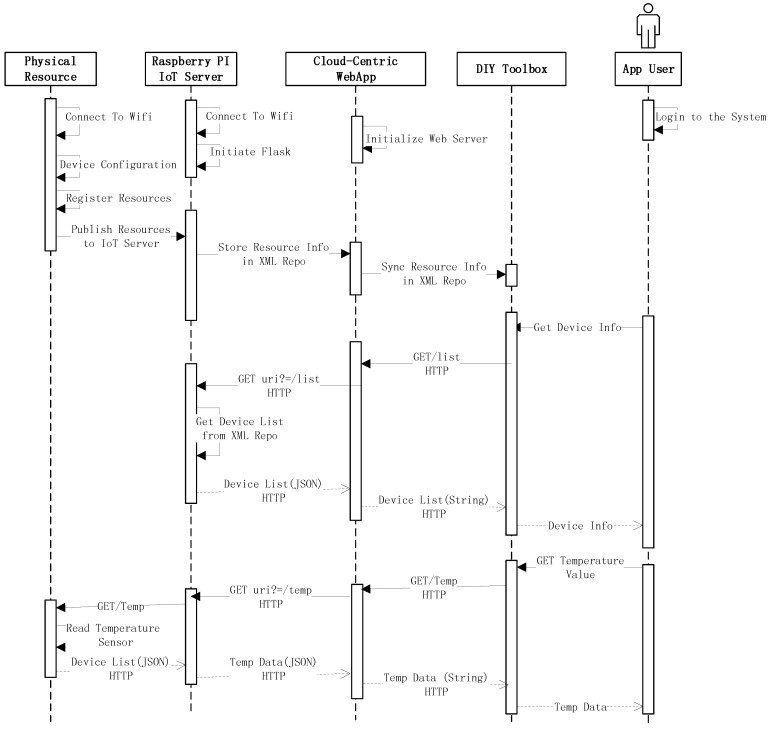
Sequence diagram of various operations within the proposed system.

Figure 5 shows the sequence of connectivity among the DIY toolbox, the cloud-based IoT application and the physical devices.

It can be perceived from the interaction model diagram that on one end, there is an actual physical device, while on the other end, the app user entity resides. The devices are associated with the IoT server, which resides on the Raspberry PI. The Raspberry PI-based webserver registers the connected devices and assigns a unique URI, which can be published to the cloud-based web application. The cloud-based web application also initializes the web server using the Flask run method. The web server receives the resources’ information in the form of XML and syncs it with the desktop-based IoT toolbox. Similarly, users can interact either with the DIY toolbox to get device information or interact with the cloud application if the user is not at his/her premises. Either way, the user is provided with the information. Therefore, in other words, if the user wants to get temperature from temperature sensor, the request will be made using either the DIY toolbox or the cloud-based IoT application, which will go all the way to the Raspberry PI-hosted IoT server. The server listens to the request and provides the data in XML format. The XML format data are converted into HTML form for rendering them in the web browser, and the end-user is informed about the operations and behavior of the sensors.

## 5. Implementation Details

In this section, an overview of the tools and technologies used in the implementation is presented and discussed. The project has three main components as shown in Figure 2. Therefore, the implementation tools have been summarized in three separate tables describing the technology stack for the respective component.

Table 1 illustrates the technology stack used for the IoT server hosted on Raspberry PI. The physical resources are associated with Raspberry PI and are implemented using the Python 3-based popular Model View Controller (MCV) framework known as Flask. Different LEDs, fan, temperature and humidity sensors have been used as physical resources and are registered in the Flask application. The application assigns a unique URI for every resource so that it can be uniquely identified by remote applications.

Table 2 depicts the development environment of the cloud-based IoT configuration repository. The repository is deployed on the AWS EC2 compute node and is developed using the Python programming language. Flask, an MVC model, is used for better project organization and the need to bypass any middleware technologies. In order to present the information in a better way, we use Bootstrap 3, which is a responsive design-based framework. The client can interact with the cloud-based configuration repository platform and requests the Raspberry PI-based IoT server using the HTTP RESTful services. The server listens to the request on the designated port and performs the action on the associated physical resource.

Table 3 summarizes the technology stack used to implement the Do-It-Yourself (DIY) toolbox. The main functionality of the system is to provide a canvas where the general public can develop their application logic by just using the dragging and dropping functionality. The wonders of windows forms are utilized. In the future, we will focus on developing this using web-based technologies in order to keep all the modules in the same technologies, mitigating the need to learn multiple technologies for the use and possible improvement of the system.

## 6. Execution Result

**Figure 6:**
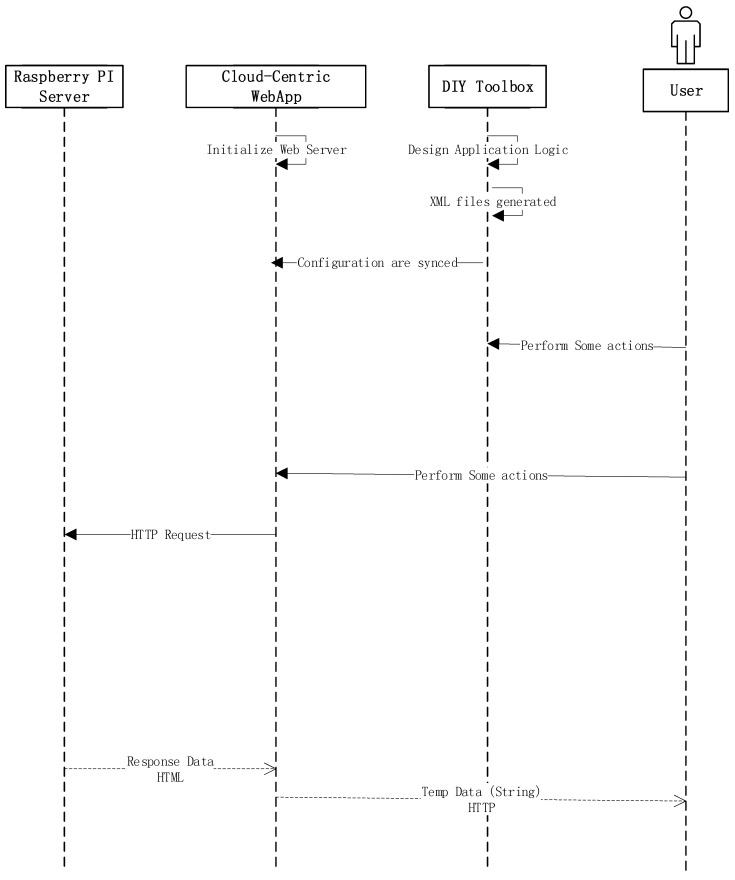
Execution sequence.

The execution sequence of the proposed system is described in Figure 6. First off, the toolbox enables application users to design the logic of the application. The toolbox provides the abstract view of the virtual device and its behavior. These virtual devices and their behaviors can be used to compose services. Once the services are composed, the configuration is stored in XML format. These configuration files are ingested by the cloud-based web-app, and the same configuration is displayed using web interfaces for better User Experience (UX). The end-users can then either interact with local configuration files or the remote cloud web interfaces to send actual commands to the IoT server, which is hosted on Raspberry PI. The web server receives the request, parses it in the respective format and sends it to the designated address.

**Figure 7:**
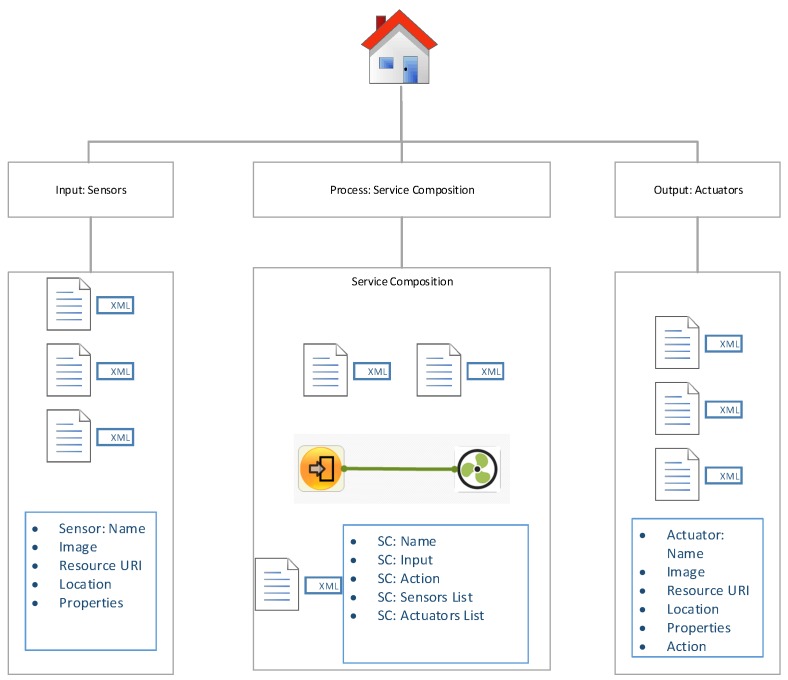
Cloud-based web application site map.

Figure 7 shows the overall sitemap and architecture diagram of the cloud-centric configuration repository web application. It takes sensors and actuator data in XML format files and represents them on web pages. Furthermore, as a result of the service composition in the DIY toolbox, the configuration settings are stored in the form of XML files. These XML files are synced on the cloud, and the web application parses these files to generate an interface that gives a better view of what is inside a particular service.

**Figure 8:**
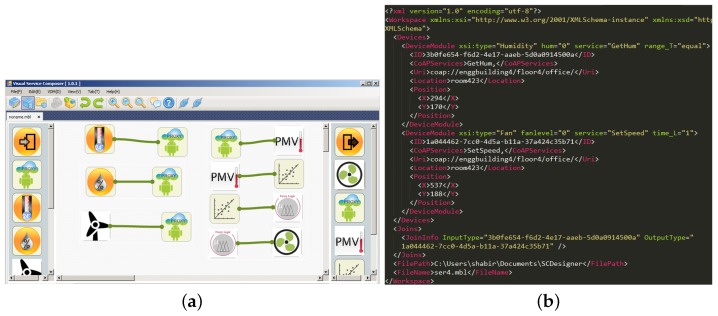
DIY toolbox service composition. (**a**) Virtual device; (**b**) XML representation.

Figure 8 shows the windows form canvas where the user can drag and drop the virtual device to compose services. The arrows represent the connection between devices. The devices at both ends are the virtual representation of physical devices that are registered with the IoT server. Once the services are composed, the configuration settings are stored in XML format, as shown Figure 8b. These are the XML files that are ingested by the cloud-based web application. The cloud-based web application consumes these files and represents the same information in a clearer and more robust way.

**Figure 9:**
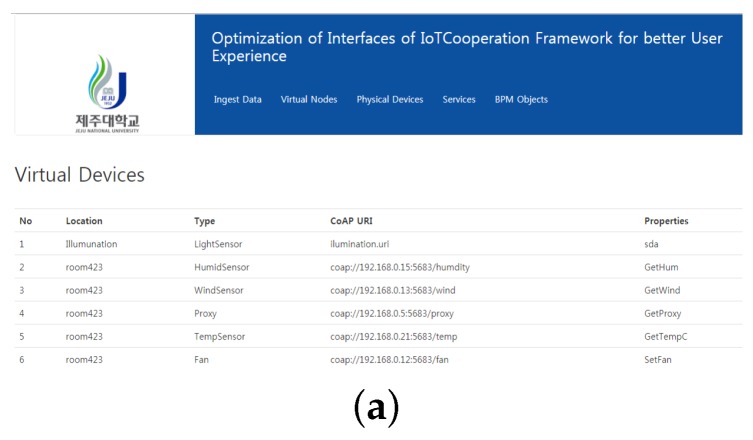

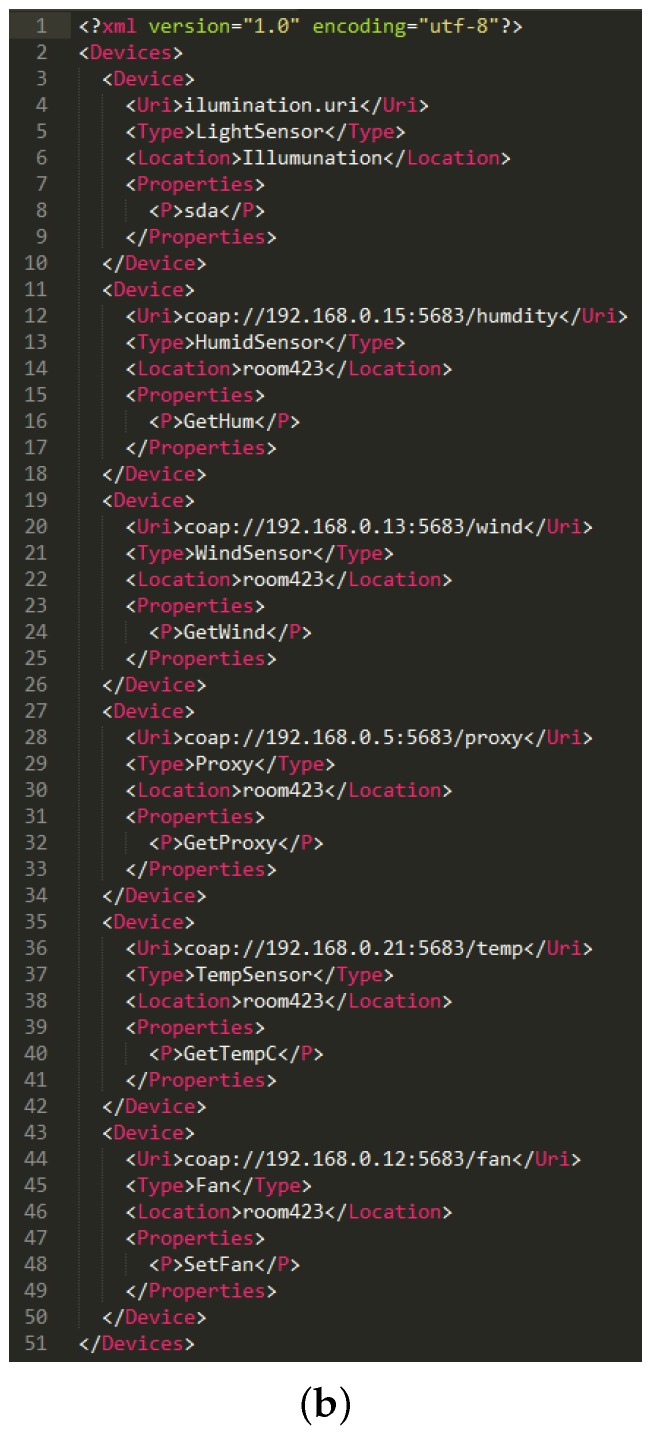
Virtual device representation. (**a**) Virtual device web interface; (**b**) XML representation.

Figure 9 shows the equivalent virtual device representation of the XML data being ingested. Every device has some attributes like the location of the device, the communication protocol, URI and the properties.

**Figure 10:**
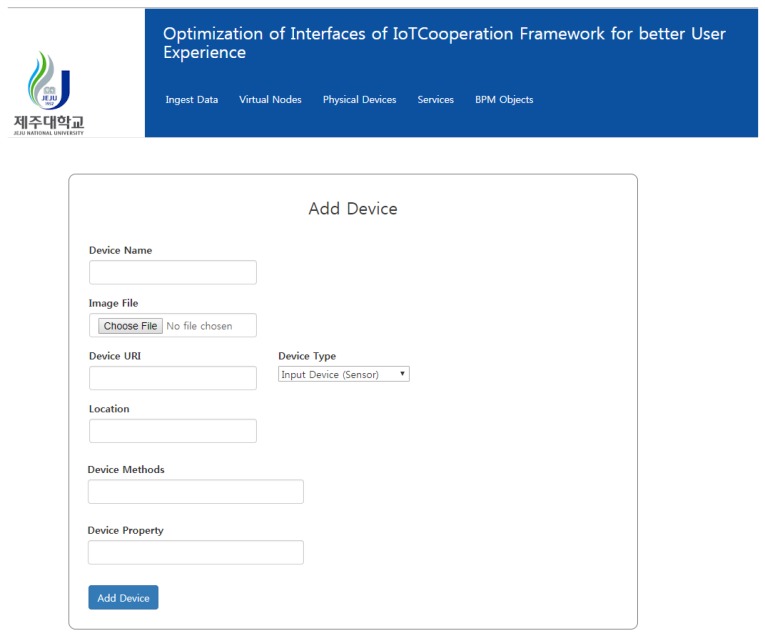
Interface for adding a device.

The devices are based on XML files that are pulled from the DIY IoT toolbox. It also has the ability to add new a virtual device using the web form shown in Figure 10.

**Figure 11:**
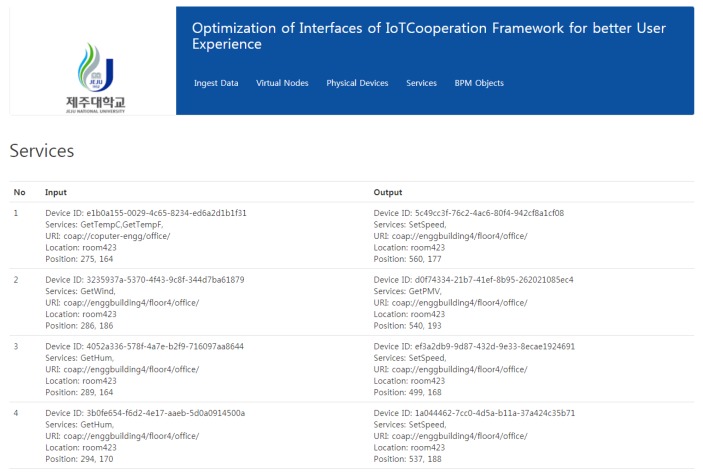
Services representation in the cloud.

Once the form is submitted, the device info is stored in the form of XML and pushed to the DIY IoT toolbox, thus keeping both the offline and cloud platform in sync. The service composition is performed on the service composition layer using simple drag and drop, as shown in the figure. Once the services are composed, the resultant XML files are pulled from the toolbox repository and represented using the web interface as shown in Figure 11.

## 7. Case Study: Smart Home

**Figure 12:**
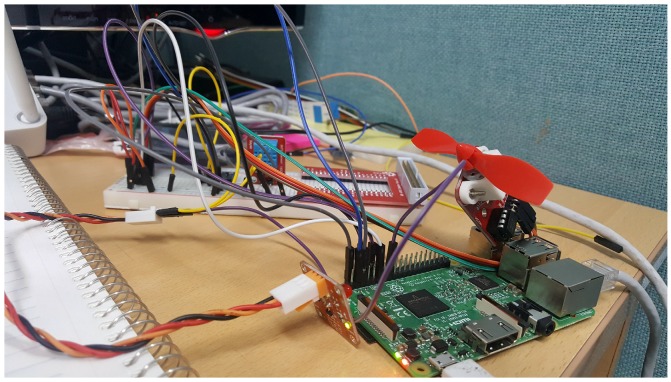
Implementation environment.

In order to evaluate the effectiveness of the proposed work, a smart home case study has been implemented as part of the experimental work. The case study also describes the way communication occurs between the cloud, IoT nodes and the DIY toolbox. Figure 12 shows the implementation environment for the case study presented in the subsequent section. The Raspberry PI 3, which acts as the IoT server, has some physical resources connected. For the smart home case study, the fan actuator, temperature sensor and light LEDs are used, which are clearly visible in the figure.

For implementation purposes, we define a generic entity, which can act as a wrapper for the smart world. The entity is named the smart space. Smart homes, smart cities and smart farms can be added as example contents.

### Add Smart Space

**Figure 13:**
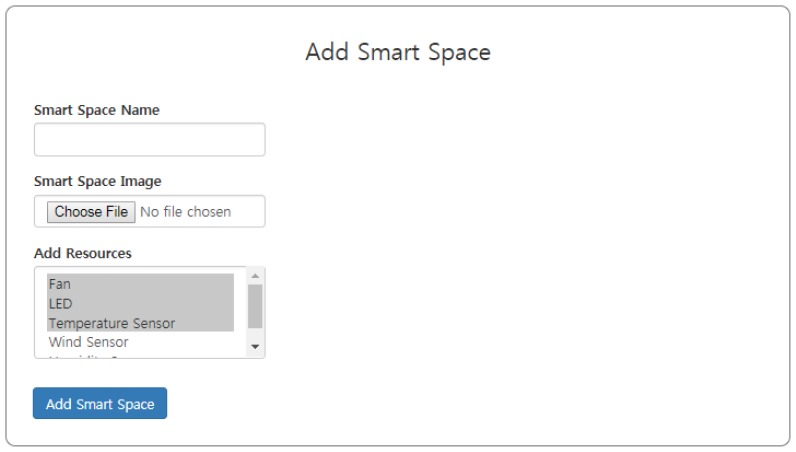
Add smart space interface.

The first step is to add a smart space to the web-based application. The smart space is a generalized content type for the smart world. The smart space could be anything like a smart home, a smart farm, etc. For the case study, the smart home is added using the web form shown in Figure 13. The name attribute of the form is the name of the smart space, for instance, the smart home in this case. The form, additionally, allows users to upload an image of their choice for the smart space. Moreover, a drop down list of virtual resources appears from which the resources can be added to the smart space. In Figure 13, the name is assigned as “smart home”, and the lights, fan and temperature and humidity sensors are added as virtual resources.

Once the smart home is added, the system presents a pictorial view of the smart home with various virtual resources added during the process described before. The resources are linked with the actual physical resources, and clicking on any of them fires a request to the IoT server. The IoT server, which is hosted on Raspberry PI, listens to the request and performs the action on the respective device.

Table 4 summarizes numerous endpoints of communication between the cloud-based IoT application and the Raspberry PI-based IoT server.

**Figure 14:**
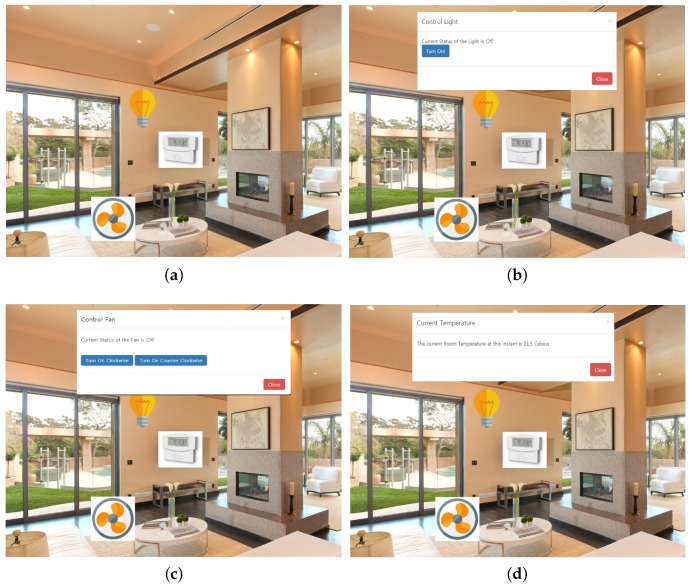
Smart home snapshots. (**a**) Smart home initial view; (**b**) Light control; (**c**) Fan control; (**d**) Temperature reading.

Figure 14 shows some snapshots of various interfaces based on the communication endpoints described in Table 4, and their respective actions are displayed in the dialog box.

Figure 14a shows the overview of the smart space. The pictorial representation of the virtual resources is presented for various locations of the home. Figure 14b illustrates the process of controlling the LED lights. The LED bulb is clicked to display a modal dialog showing the current status of the LED and the option to change the status. In the same way, in Figure 14c, the dialog box that pops up as a result of clicking on the fan icon is presented. The fan can be turned on in the clockwise direction or in the counter-clockwise direction. If the current status is turned on, a button will appear instead, which will turn off the fan. Lastly, in Figure 14d, the temperature sensor icon is clicked to get the current room temperature from the sensor.

## 8. Comparison and Significance

As discussed in the earlier section of the paper, to the best of the authors’ knowledge, this work is a novel attempt towards the cloud-based DIY toolbox. Many similar applications exist, but they are either fully web based or fully desktop based. In the proposed work, the compute node service of the cloud platform is utilized in order to boost the performance of the web application, and the specialized cloud IoT is not considered, because the authors believe it to be overkill in this scenario. In order to assess the effectiveness of the proposed system, a comparative analysis of the system with the related tools discussed in the Related Work Section has been carried out. The result has been summarized in Table 5. The features that play a vital role for any application are considered for this study. It has been shown in the table that the proposed system outperforms the related tools in many aspects. It can be seen from the table that Node-Red is a somewhat similar approach. The main difference is that Node-Red renders blocks of code visually and combines these blocks to form flow of the program, which means the DIY support is partial and to some extent technical expertise is required. Another application named SAM also performs similarly. The main problem is that the program is inherently written for a specific platform and thus will not work if the underlying platform changes. Moreover, the DIY toolboxes, like OPEL Software Platform and Super Stream Collider, use web-based APIs to semantically model the behavior of the IoT application, but these applications cannot be accessed offline. Similarly, Particle.IO and Dweet.IO are platforms as a service and fully cloud based, so they do not give much flexibility to the general public.

In this work, the concept of the generic entity, known as the smart space, is introduced, and a smart home-based case study has been implemented as a proof of concept. The generic entity can be extended to scenarios like smart cities and smart farms, which also supports the significance of the work. Moreover, the demands to have an application that can work offline using traditional desktop applications, that can work online from remote places and that supports different protocols are increasing exponentially, and this paper is the first attempt to address all of the above-mentioned issues.

## 9. Conclusions

This paper outlines the procedure for the design and implementation of a novel architecture for enabling the general public to build IoT applications with zero coding. The configuration settings are stored in XML format and are ingested by the cloud-based web application for better UX and remote connectivity. For the IoT server, Raspberry PI is used, and the powerful API of Flask MVC has been leveraged. Resources are represented in XML format and are kept in sync between the DIY toolbox and the cloud-based web application. End-users can either use the toolbox on premises or use cloud-based web application to remotely interact with the smart space. The novelty of the presented work is the distribution of configuration settings both in local storage and cloud storage, thus providing users with the flexibility to remotely access the application even if the users are not at their premises. A smart home case study has been implemented for the evaluation of the significance of the proposed work, and the various interfaces have been discussed in detail. The significance of this work has been outlined with a benchmark study of the proposed system with similar tools, and it has been shown that the system performs better with respect to the existing tools. The future direction of this project can be to allow users to model and design the application logic using web-based technologies like HTML5 canvas and drawing tools, thus making both systems imitate the majority of their functions.

## Figures and Tables

**Table 1:** Technology stack of the IoT server. IDE, Integrated Development Environment.

Component	Description
Hardware	Raspberry PI 3 Model B
Operating System	Raspbian
Memory	1 GB
Server	Flask Webserver
Resources	LED, Fan, Temperature Sensor, Humidity Sensor, Breadboard, Connecting Wires
Libraries	General Purpose Input/Output (GPIO), Untangle for XML Parsing, Jinja Template
IDE	Vim, PyCharm (Remote Access)
Programming Language	Python 3

**Table 2:** Technology stack of the cloud-based web application.

Component	Description
Operating System	Linux AWS EC2 Compute Node
IDE	Sublime Text 3, PyCharm
Programming Language	Python, Flask, JavaScript, HTML, CSS
Libraries and Framework	Untangle for Parsing XML, Bootstrap3, Jinja3 for Templating
Core Programming Language	Python 3
Browser	Chromium, Google Chrome, Firefox, Safari
Server	Flask-based Webserver
Persistence	XML Repository in Sync with the DIY Toolbox

**Table 3:** Technology stack of the DIY toolbox.

Component	Description
Operating System	Windows 8, 64 bits
CPU	Intel (R) Core(TM) i5-4570 CPU @3.20 GHz
Primary Memory 12 GB IDE	Visual Studio 2017, Community Version
Programming Language	C Sharp
Framework	.Net Framework 4.5
Libraries	Windows Form, KRBTabControl for implementing Control tabs
Persistence	XML Files in Sync with the Cloud App

**Table 4:** Communication endpoints.

URL	Action
http://rasp-localhost/fan?op=on&dir=clk	Turn on the fan in the Clockwise Direction
http://rasp-localhost/fan?op=on&dir=anticlk	Turn on the fan in the Counter-Clockwise Direction
http://rasp-localhost/fan?op=off	Turn Off the Fan
http://rasp-localhost/led?op=on	Turn On the LED Light
http://rasp-localhost/led?op=off	Turn Off the LED Light
http://rasp-localhost/get-sensor-data	Get the Current Temperature from the Temperature Sensor

**Table 5:** Comparative analysis of the proposed system with the related tools.

No	Name	Open-Source	DIY Support	Remote Access	Configuration Repository	Offline Access	Cloud Support	Application Type	Generic Entity Support	Programming Language
1	SAM	No	Partial	No	XML	Yes	Yes	Desktop	No	JavaScript/Python
2	Super Stream Collider	No	Partial	Yes	JSON/PLSQL	No	No	Web	No	JavaScript
3	OPEL	Yes	No	Yes	JSON	No	No	Mobile/Web	No	JavaScript
4	Node-red	Yes	Partial	Yes	JSON	No	No	Web	No	Node.js
5	Glue.thing	No	Yes	Yes	JSON	No	No	Web	No	Node.js
6	Thing.work									
7	OpenIoT	Yes	No	Yes	XML	No	Yes	Web	No	Java
8	Particle.IO	No	No	Yes	XML	No	Yes	Middleware	Yes	Java
9	Dweet.IO	Yes	No	No	JSON	No	No	Middleware	No	Multi-language Support
10	Proposed System	Yes	Yes	Yes	XML	Yes	Yes	Hybrid (Web and Desktop)	Yes	Python
